# Elamipretide mitigates ischemia-reperfusion injury in a swine model of hemorrhagic shock

**DOI:** 10.1038/s41598-023-31374-5

**Published:** 2023-03-18

**Authors:** N. Patel, M. A. Johnson, N. Vapniarsky, M. W. Van Brocklin, T. K. Williams, S. T. Youngquist, R. Ford, N. Ewer, L. P. Neff, G. L. Hoareau

**Affiliations:** 1 Department of Surgery, Wake Forest Baptist Medical Center, Winston-Salem, NC USA; 2 Department of Emergency Medicine, University of Utah, Salt Lake City, UT USA; 3 Department of Pathology, Microbiology, and Immunology, University of California-Davis, Davis, CA USA; 4 Department of Surgery, University of Utah, Salt Lake City, UT USA; 5 Department of Vascular/Endovascular Surgery, Wake Forest Baptist Medical Center, Winston-Salem, NC USA; 6 Department of Pediatric Surgery, Wake Forest Baptist Medical Center, Winston-Salem, NC USA; 7 Nora Eccles-Harrison Cardiovascular Research and Training Institute, Salt Lake City, UT USA

**Keywords:** Trauma, Metabolic disorders, Acute inflammation

## Abstract

ischemia-reperfusion injury (IRI) after hemorrhage is potentiated by aortic occlusion or resuscitative endovascular balloon occlusion of the aorta (REBOA). Given the central role of mitochondrial injury in shock, we hypothesized that Elamipretide, a peptide that protects mitochondria, would mitigate IRI after hemorrhagic shock and REBOA. Twelve pigs were subjected to hemorrhagic shock and 45 min of REBOA. After 25 min of REBOA, animals received either saline or Elamipretide. Animals were transfused with autologous blood during balloon deflation, and pigs were resuscitated with isotonic crystalloids and norepinephrine for 4.25 h. Elamipretide-treated animals required less crystalloids than the controls (62.5 [50–90] and 25 [5–30] mL/kg, respectively), but similar amounts of norepinephrine (24.7 [8.6–39.3] and 9.7 [2.1–12.5] mcg/kg, respectively). Treatment animals had a significant reduction in serum creatinine (control: 2.7 [2.6–2.8]; Elamipretide: 2.4 [2.4–2.5] mg/dL; *p* = 0.04), troponin (control: 3.20 [2.14–5.47] ng/mL, Elamipretide: 0.22 [0.1–1.91] ng/mL; *p* = 0.03), and interleukin-6 concentrations at the end of the study. There were no differences in final plasma lactate concentration. Elamipretide reduced fluid requirements and protected the kidney and heart after profound IRI. Further understanding the subcellular consequences of REBOA and mitochondrial rescue will open new therapeutic avenues for patients suffering from IRI after hemorrhage.

## Introduction

Ischemia-reperfusion injury (IRI) is a common clinical sequela for many pathologic insults, including hemorrhage, trauma, sepsis, myocardial infarction, stroke, and cardiac arrest^1–4^. While many experimental strategies (e.g., ischemic, mechanical, and pharmacological pre-, per-, and post-conditioning) to treat the ensuing mitochondrial dysfunction are emerging, pharmacologic adjuncts like vasopressors remain the most often employed interventions to counteract the deranged physiology of IRI. Unfortunately, such measures augment hemodynamics at the macro level while failing to address the dysfunction at the cellular level^5–10^.

Traditional management of IRI often employs the usual critical care adjuncts of volume expansion with isotonic crystalloids and vasomotor tone support with vasopressors. However, in profound IRI-induced vasoplegia and distributive shock, the high resuscitation requirements may further contribute to complications such as fluid overload, acute kidney injury, pulmonary edema, abdominal compartment syndrome, and coagulation dysfunction^11–17^. This has been particularly well studied in preclinical myocardial injury models. But pilot clinical trials have not been translated into practice^5,8^. While in the newer area of research, hemorrhagic shock, various pharmacologic agents to lessen IRI have been studied, none have translated into routine clinical use^18–22^.

With the recent enthusiasm for endovascular techniques for resuscitation in trauma, there is a growing awareness of the profound IRI that often follows even short periods of aortic occlusion. Despite the increasing evidence of survival benefit with resuscitative endovascular balloon occlusion of the aorta (REBOA) used in the proper patient population^23,24^, there is a need to mitigate REBOA-associated IRI to fully realize its benefits. To date, most IRI mitigation efforts have focused on refining balloon manipulation to control distal perfusion during active resuscitation. While partial or intermittent balloon inflation techniques limit IRI severity^25,26^, REBOA is still primarily relegated to high-volume trauma centers^27^ and faces clinical adoption and implementation challenges. Therefore, renewed efforts to mitigate IRI by other means (e.g., novel therapeutics for endovascular technologies) are necessary to catalyze this potential paradigm shift in resuscitation. Various compounds, such as valproate, hydrogen sulfide, and, more recently, the combination of adenosine, lidocaine, and magnesium^28–30^ have been studied with limited success. Moreover, none of these medications have been used clinically to mitigate post-REBOA IRI. One reason may be that their mechanisms of action were not targeting the correct cellular processes.

Mitochondria are the cell's power plants and represent critical mediators in the initiation and propagation of IRI. Injury to the mitochondrial structure has been reported following trauma^31,32^, hemorrhagic shock^33^, and aortic occlusion^34^. Mitochondrial dysfunction plays an essential role in critically ill patients immediately following injury and is also associated with delayed organ failure and death. Because critically ill trauma patients often fall victim to the compounding effects of trauma, hemorrhagic shock, and even sepsis, this organelle represents an important therapeutic target for novel therapies.

Such therapies that can reduce mitochondrial dysfunction in patients suffering from IRI may likely improve morbidity and mortality. To date, there is little understanding of the extent of mitochondrial injury after an extreme resuscitation therapy like REBOA or whether pharmaceutical interventions to preserve mitochondrial function can minimize the associated IRI. Small peptides have been developed specifically to improve mitochondrial function in children with inherited mitochondrial diseases and may offer a rapidly translatable therapy. Elamipretide is one such compound that selectively binds to cardiolipin within the mitochondrial membrane to prevent the loss of mitochondrial cristae structure and organization^35^. Elamipretide also protects mitochondrial bioenergetics by inhibiting cytochrome-c peroxidase activity and mitochondrial permeability transition pore opening, preventing apoptosis and oxidative stress^36^.

Elamipretide has improved mitochondrial function in both the heart and kidneys in large animal studies^36,37^. For example, in a rabbit model of coronary ischemia-reperfusion, Elamipretide administered before reperfusion reduced myocardial infarct size^38^. Similarly, Elamipretide reduced mitochondrial and renal injury in humans with renovascular hypertension undergoing reperfusion following percutaneous transluminal renal angioplasty^39^.

We sought to rescue mitochondrial function in a pig model of hemorrhagic shock and REBOA-induced IRI and to quantify the effect of Elamipretide on such a model. We hypothesized that Elamipretide treatment would reduce REBOA-associated IRI, as evidenced by a reduction of systemic biomarkers of ischemia by the end of the study.

## Methods

### Overview

**Figure 1:**
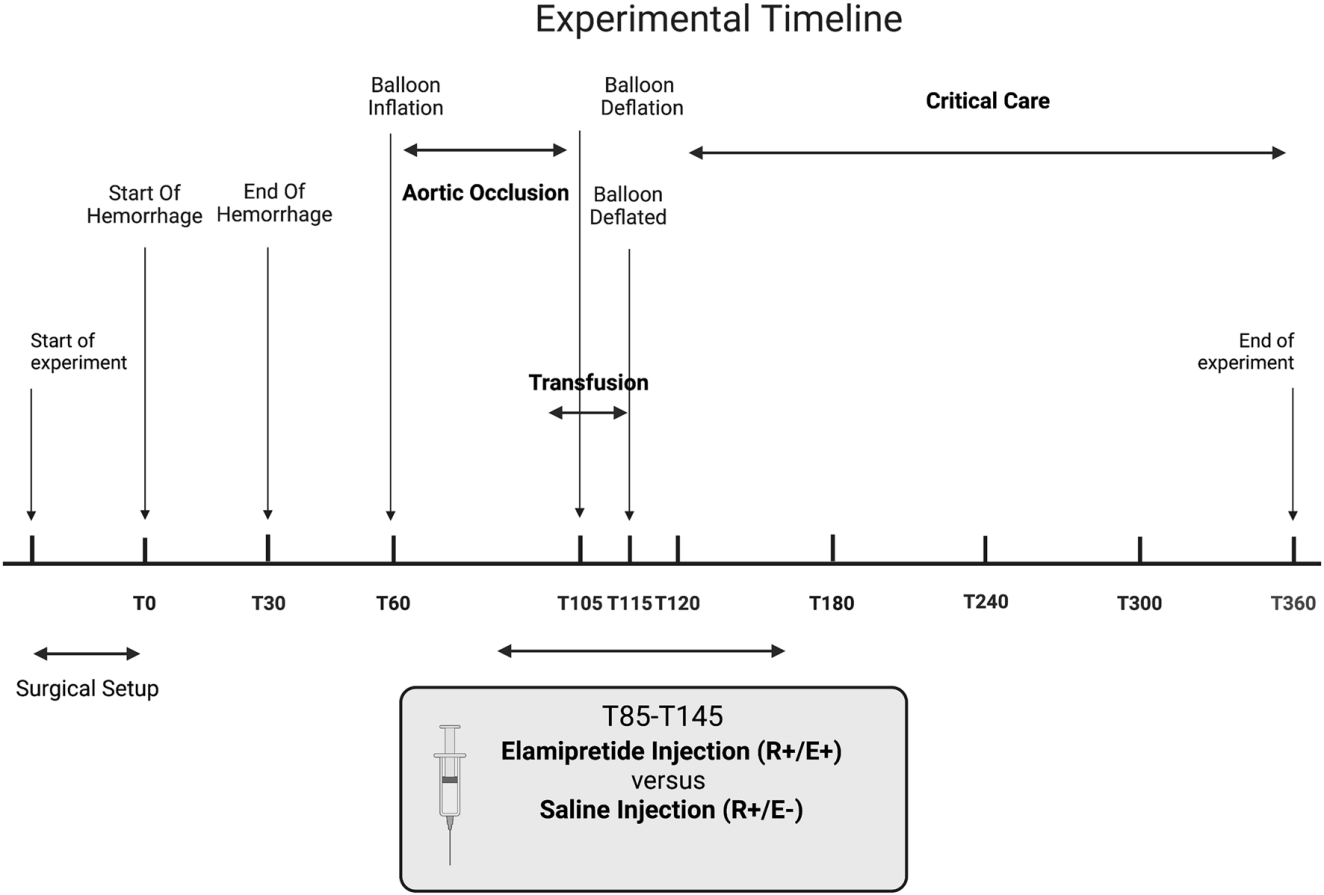
Experimental overview.

This study was approved by the institutional animal care and use committee at the University of Utah (approval number 19-11,002). All experiments were performed in accordance with relevant guidelines and regulations. The animal research: reporting of in vivo experiments guidelines (ARRIVE) were used to prepare this manuscript. An overview of the study is provided in Fig. 1.

### Animal preparation

Castrate male Yorkshire swine (*Sus scrofa*; Premier BioSource) or non-pregnant females weighing 59–84 kg (6–8 months old) were acclimatized for at least 7 days before experimentation in temperature- and light-controlled pens with access to environmental enrichment. They were fasted for 8–12 h before anesthesia. Anesthesia was induced with an intramuscular injection of ketamine (2.2 mg/kg, Vedco, Saint Joseph, MO) and xylazine (2.2 mg/kg, Vedco, Saint Joseph, MO), and inhaled 5% isoflurane (Dechra, Northich, United Kingdom), as needed. After endotracheal intubation, gaseous isoflurane anesthesia was maintained at 1–2.5% in 2 L/min of oxygen. Animals were mechanically ventilated with a fraction of inspired oxygen titrated to maintain a pulse oximetry reading > 95%, positive end-expiratory pressure of 4 cm H_2_O, tidal volumes of 6–8 mL/kg, and a variable respiratory rate to maintain an end-tidal CO_2_ of 35–45 mmHg. Warmed balanced isotonic fluids (Plasmalyte 148, 140 mEq sodium, 5 mEq potassium, 3 mEq magnesium, 98 mEq chloride, 27 mEq acetate, and 23 mEq gluconate, Baxter, Deerfield, IL) were administered intravenously at 5 mL/kg/h. Throughout the experiment, hypoglycemia, hyperkalemia, and ionized hypocalcemia were treated as needed. Animals were kept on warming blankets to maintain normothermia.

### Vascular access and surgical preparation

After local infiltration with lidocaine, vascular access was established percutaneously with the Seldinger technique under ultrasound guidance. A 9 Fr multi-lumen access catheter was inserted in a jugular vein to deliver autologous blood. A triple-lumen catheter was inserted through the sheath for medication infusions and central venous pressure monitoring. A 5 Fr carotid artery catheter was placed for proximal blood pressure monitoring. A 7 Fr sheath catheter was placed in a femoral vein for isotonic crystalloid boluses. A 12 Fr sheath catheter was placed into a femoral artery for controlled hemorrhage, blood sampling, and to introduce a custom-made 7 Fr REBOA catheter. The REBOA catheter was positioned with the balloon immediately superior to the diaphragm, which was confirmed by fluoroscopy. A 7 Fr sheath catheter was introduced in the contralateral femoral artery for distal blood pressure monitoring. A 20 Fr Foley catheter was placed in the bladder using a suprapubic surgical approach, and the spleen was removed via a midline laparotomy to prevent auto-transfusion.

### Data collection

The goal mean arterial pressure (MAP) before starting experimental procedures was 65 mmHg. Animals were given up to two 5 mL/kg bolus of Plasmalyte 148 if the MAP was < 65 mmHg. MAP above and below the balloon, as well as central venous pressure, were continuously recorded (PowerLab data acquisition platform, ADInstruments, Colorado Springs, CO). Arterial blood was sampled, and urine output was quantified at predetermined intervals. We also calculated the total fluid balance [Fluid bolus (mL/kg)—Urine output (mL/kg)].

### Experimental flow (Fig. 1)

Hemorrhagic shock was induced by removing 25% of the animal's estimated blood volume [25% × 60 (mL/kg) × body weight (kg); blood losses during the experimental setup were subtracted from the volume of blood to be removed] over 30 min through the femoral arterial line. Removed blood was collected in citrated blood collection bags under constant agitation and then stored in a warm water bath at 38 °C.

Following the 30-min hemorrhage phase (T30), animals remained untreated for an additional 30 min (T60). At T60, animals were randomized to one of two groups using block randomization with blocks of random size: control with the administration of placebo (0.9% saline, Baxter, Deerfield, IL) or Elamipretide (BOC Sciences, Shirley, NY) administration (N = 6/group). In both groups, animals underwent 45 min of complete aortic occlusion (from T60 until T105). Complete occlusion was continuously confirmed via loss of pulsatility on the distal arterial waveform. Animals in the Elamipretide group received 0.1 mg/kg of Elamipretide intravenously over 1 h, starting 20 min before balloon deflation. This dosing regimen was based on previously published data^37,38^.

### Critical care

At T100, animals in both groups were transfused with their shed blood over 15 min. Simultaneously, an intravenous norepinephrine (Baxter, Deerfield, IL) infusion was initiated at 0.02 mcg/kg/min, and intravenous calcium (1.6 g of calcium gluconate, VetOne, Boise, ID) was administered to prevent citrate-induced hypocalcemia. At T105, the balloon was deflated over 10 min, and the REBOA catheter was removed. Animals then entered the critical care phase, where they were resuscitated with a combination of balanced isotonic crystalloids (Plasmalyte 148) and norepinephrine to maintain a MAP > 65 mmHg according to a pre-specified critical care algorithm (see Supplemental Fig. 1).

### Inclusion and exclusion criteria

Pigs with a white blood cell count > 25.0 × 10^9^/mL, serum creatinine concentration > 2.3 mg/dL, aspartate aminotransferase activity > 47 U/L, or alanine aminotransferase activity > 55 U/L at baseline were excluded from the study. Pigs were also excluded if MAP remained < 65 mmHg despite up to two 5 mL/kg boluses of isotonic crystalloids and down-titration of isoflurane during the setup phase.

### Biological samples

At the end of the experiment, representative sections of the heart, duodenum, liver, and kidney from each animal (N = 6 Elamipretide-treated and N = 6 controls) were placed in 10% buffered formalin for subsequent histopathological assessment. Additionally, 2 mm core biopsies of the left ventricle and kidney (cortex and medulla) from each animal were immediately placed in 1% osmium tetroxide and fixed overnight at 4 °C for electron microscopy assessment.

### Histopathology and electron microscopy

Formalin-fixed samples of the liver, heart, kidney, and duodenum were sliced, processed, sectioned, and stained with hematoxylin and eosin according to a routine protocol. A board-certified veterinary anatomic pathologist (NV) evaluated and graded 2 to 3 sections/tissue for any evidence of cell death, ischemic necrosis, or inflammation. For each organ, a grading scheme of 0–3 was assigned. Grade 0 was consistent with no pathology. Grade 1 was assigned to tissue demonstrating minimal pathology, including rare cell death, focal inflammation, or ischemic necrosis. Grades 2 and 3 were assigned in cases of moderate and more severe pathology, respectively. All grading was performed blindly without prior knowledge of study group assignment.

Osmium-fixed specimens were rinsed in a buffer and postfixed in 2% osmium tetroxide for 1 h at room temperature. They were then rinsed in deionized water and pre-stained with uranyl acetate for 1 h at room temperature. The samples were then dehydrated in graded ethanol series and 3 times in pure acetone and then embedded in epoxy resin. Seventy nm sections were post-stained with uranyl acetate for 10 min and lead citrate for 5 min. Sections were evaluated using JEM-1400 plus (JEOL, Tokyo, Japan) transmission electron microscope with a CCD Gatan camera (University of Utah Core facility). Mitochondrial size and shape were quantified using the ImageJ “Analyze Particles” feature. The following mitochondrial parameters were recorded: area (μm^2^), major and minor axes (μm), and aspect ratio (major/minor axis). Additionally, mitochondrial morphology elements were assessed by a board-certified pathologist blinded to the group assignment. Specifically, in the myocardial samples, mitochondria were evaluated for cristae integrity, intactness of the outer and inner membranes, overall shape and outline of the mitochondria, presence of peri mitochondrial clearing, matrix aggregation, and intactness and frequency of inter mitochondrial junctions, as previously described^40^. Grade 0–3 were assigned to electron micrographs evaluated collectively for each capture (a total of 4, 16, and 16 micrographs were evaluated at 1000× , 2000× , and 4000× magnification, respectively) (Supplemental Fig. 2).

### Serum analysis

Anticoagulated whole blood was used immediately to measure plasma lactate concentration over time and creatinine concentrations at T0 and T360 (iStat, Abbott, Chicago, IL). Serum troponin was measured (Siemens Immulite 20 0 0, Tarry-town, NY) at T0 and T360 using a commercial veterinary laboratory (University of California-Davis, School of Medicine, Davis, CA). Serum samples were also used to measure serum cytokine concentrations (Luminex Discovery Assays, R&D Systems, LXSAPM, Austin, TX). First, serum samples were thawed on ice, vortexed, and cleared at 1000 g for 10 min at 4 °C. Samples were diluted 2× with the diluent provided in the kit. Then, 25 µL of each sample were added to a 96-well assay plate and incubated with antibody-conjugated magnetic multiplexing beads for 2 h at room temperature on a microplate shaker at 800 rpm. Following a wash step using a magnetic plate washer, captured analytes were then probed under similar conditions with biotin-labeled detection antibodies for 1 h and washed before treatment with Strepdavidin-PE for another 30 min. Following the final wash steps, the beads were re-suspended in a reading buffer before running the plate on the Luminex MAGPIX platform.

### Data analysis

Our primary outcome was serum lactate concentration at the end of the study. Secondary outcomes included the volume of isotonic crystalloids and norepinephrine dose required to maintain a target MAP of 65–75 mmHg and tissue injury evidenced by histologic and electron microscopy injury scores as well as final serum troponin and creatinine concentrations for the heart and kidney, respectively. Data are presented as mean + /− standard deviation or median [interquartile range] depending on data distribution. Between groups comparisons were made using a t-test or a Mann–Whitney U test for statistical significance (with adjustment for repeated measures when appropriate). Significance *p*-value was set at < 0.05. GraphPad Prism 9.2.0 was used to generate mean Log2 fold changes in serum cytokines from baseline and graph pg/mL concentrations over time. Statistically significant differences between control and Elamipretide-treated cohorts over time were assessed using a 2-tailed t-test assuming unequal variances. The sample size was based on heuristics of prior studies, using a two-tailed t-test with a significance of 0.05, power of 80%, and an estimated effect size of 2 (reflecting a difference in serum lactate of 3 mmol/L between groups and a standard deviation of 1.5 mmol/L).

### Ethics approval and consent to participate

This study was approved by the institutional animal care and use committee at the University of Utah (approval number 19–11002) and by the Animal Care and Use Review Office of the US Army Medical Research and Materiel Command.

## Results

### Pre-intervention data

No animals were excluded from the study. There was no difference between groups in baseline biochemical parameters, white blood cell and platelet counts, or urine output (See Table 1). While there was no difference in baseline parameters between groups, MAP was statistically higher in the Elamipretide group immediately before aortic occlusion (See Table 2). There was no other difference between groups during the occlusion phase.

**Table 1:** Baseline characteristics.

Median [Interquartile Range]	Control (N = 6)	Elamipretide (N = 6)	*P* Value
Male/Female	1/5	2/4	0.50
Weight (kg)	76.0 [70.0–79.2]	77.2 [74.4–79.0]	0.87
pH	7.43 [7.40–7.47]	7.45 [7.44–7.49]	0.42
pCO_2_ (mmHg)	44.6 [41.1–49.7]	42.7 [40.2–44.2]	0.30
pO_2_ (mmHg)	150.0 [131.0–160.0]	156.0 [138.0–175.0]	0.75
Bicarbonate (mmol/L)	30.0 [28.3–32.4]	30.0 [29.1–30.8]	1.0
Base Excess (mmol/L)	5.0 [4.0–9.0]	6.0 [5.0–7.0]	0.57
Lactate (mmol/L)	2.4 [1.7–2.5]	2.5 [2.3–3.0]	0.52
Glucose (mg/dL)	121.5 [108.0–174.0]	107.0 [105.0–110.0]	0.23
Blood urea nitrogen (mg/dL)	4.5 [4.0–10.0]	4.5 [4.0–7.0]	0.74
Creatinine (mg/dL)	1.6 [1.5–1.8]	1.6 [1.3–1.7]	0.37
Hematocrit (%)	30 [28–31]	31 [28–32]	0.68
Hemoglobin (g/dL)	10.2 [9.5–10.5]	10.4 [9.5–10.9]	0.68
White blood cell count (10^9^/L)	13.3 [12.2–14.3]	14.2 [13.4–15.9]	0.47
Platelet Count (10^9^/L)	262 [248–317]	215 [198–241]	0.14
Urine Output (mL/kg)	1.28 [0.8–2.28]	3.49 [1.69–4.63]	0.08
Mean arterial blood pressure (mmHg)	87.6 [77.8–96.0]	104.2 [98–111.0]	**0.03**

Significant values are in bold.

**Table 2:** Hemodynamic parameters over time. 1000 Hz sampled data is taken as an average during the period of interest.

Mean (standard deviation)	Control (N = 6)	Elamipretide (N = 6)	*P* value
End of hemorrhage proximal blood pressure (mmHg)	35.98 (4.27)	43.83 (8.89)	0.065
Distal mean arterial blood pressure during hypotension (mmHg)	36.96 (5.22)	46.32 (6.46)	**0.041**
Proximal mean arterial blood pressure during aortic occlusion (mmHg)	130.00 (8.66)	133.33 (15.75)	0.699
Distal mean arterial blood pressure during aortic occlusion (mmHg)	21.75 (4.01)	22.35 (4.32)	0.937
Proximal mean arterial blood pressure during critical care (mmHg)	67.12 (4.93)	77.62 (6.15)	**0.026**
Mean central venous pressure during critical care (mmHg)	12.19 (5.80)	8.19 (1.22)	0.093

Significant values are in bold.

### Critical care data

**Figure 2:**
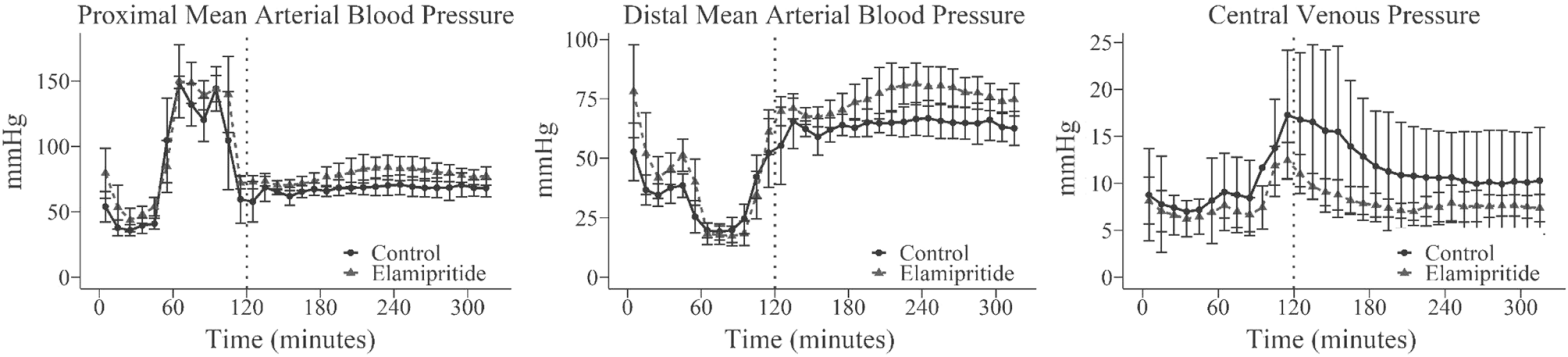
Proximal arterial, distal arterial, and central venous blood pressure over time. 10-min bins are represented as mean with standard deviation as error bars. The dotted vertical line denotes the removal of the aortic balloon and the start of the critical care phase.

**Figure 3:**
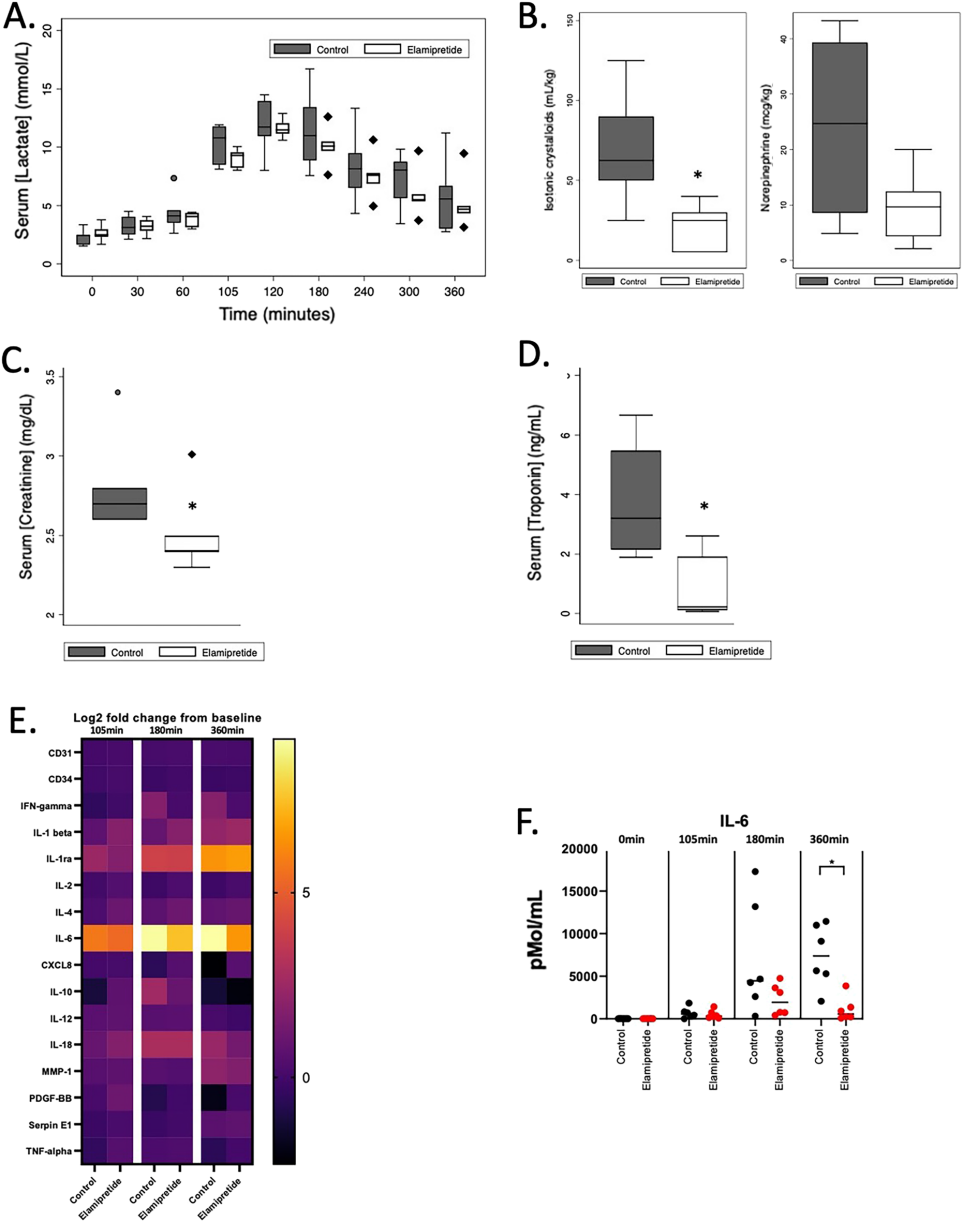
(**A**)**.** Serum lactate concentrations over time between groups. (**B**)**.** Total volume of weight-adjusted isotonic crystalloid bolus required to maintain normotension. Cumulative norepinephrine dose required to maintain normotension at the end of the experiment. (**C**)**.** Serum Creatinine and (**D**)**.** serum troponin at the end of the study. Horizontal line = median; upper and lower limits of each box = interquartile range. Whiskers = 5–95% range. Data points outside of this range are plotted as individual points. (**E**)**.** Log2 fold changes in serum cytokines concentrations compared to baseline. (**F**)**.** Serum interleukin 6 concentration over time.

During the subsequent critical care phase, the average MAP was significantly higher in the Elamipretide group compared to controls, *p* = 0.026 (Fig. 2). Serum lactate concentration confirmed that animals in this model consistently developed a significant IRI, as evidenced by peak serum lactate concentrations at T120 of > 10 mmol/L (Fig. 3A). We found no difference in final serum lactate concentrations between the two groups (control: 5.7 [3.0–6.7]; Elamipretide: 4.7 [4.3–5.0] mmol/L; *p* = 0.70). Elamipretide treatment significantly reduced the dose of isotonic crystalloids required to maintain normotension (control: 62.5 [50–90] mL/kg, Elamipretide: 25[5–30] mL/kg) (*p* = 0.01), while there was no difference in the total amount of norepinephrine required for either group (Fig. 3B).

### Biochemical analysis

There was an expected rise in serum creatinine concentration from baseline in both groups (control: 1.0 [1.0–1.3]; Elamipretide: 1.0 [0.7–1.1] mg/dL; *p* = 0.42) at the end of the injury, indicating that both groups suffered acute kidney injury per Kidney Disease Improving Global Outcomes guidelines^41^. However, treatment with Elamipretide during resuscitation significantly reduced serum creatinine concentration (control: 2.7 [2.6–2.8]; Elamipretide: 2.4 [2.4–2.5] mg/dL; *p* = 0.04) at the end of the study, despite lower crystalloid fluids requirements (Fig. 3C). Furthermore, while there was no difference in urine output throughout the study between groups (control: 10.8 [8.7–12.5]; Elamipretide: 17.1 [14.7–18.2] mL/kg; *p* = 0.09), the total fluid balance was significantly reduced in the Elamipretide group (with some pigs presenting a negative fluid balance), as expected with the reduced crystalloids requirement (control: 53.3 [41.3–79.8]; Elamipretide: 3.4 [− 1.5 to 11.8] mL/kg; *p* =  < 0.01). Elamipretide significantly reduced final (T = 360) serum troponin concentration (control: 3.20 [2.14–5.47] ng/mL, Elamipretide: 0.22 [0.1–1.91] ng/mL; *p* = 0.03) (Fig. 3.D). There was a significant reduction in serum interleukin 6 concentration at the end of the experiment in the Elamipretide group (*p* =  < 0.01) (Fig. 3E, F, Supplemental Fig. 3).

### Histologic analysis and electron microscopy

**Figure 4:**
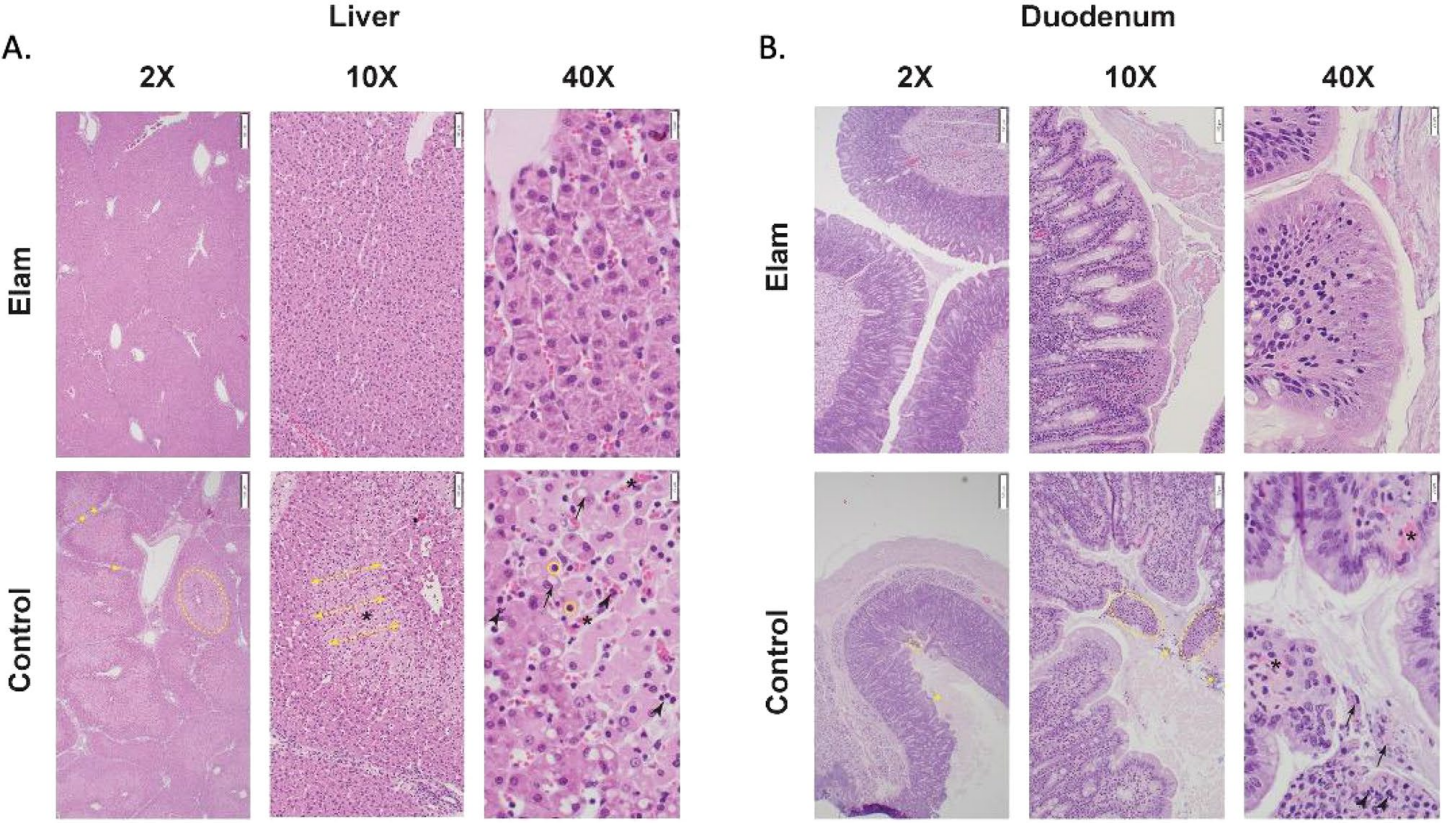
(**A**)**.** Histologic liver sections from control and Elamipretide-treated pigs presented at various magnifications. At 2× magnification, there is evidence of centrilobular to midzonal necrosis characterized by hepatocytes pallor (dashed yellow ellipse) in the control pigs but not in the Elamipretide-treated pigs. Also, interlobular space is widened in controls due to lobular collapse and edema. At 10× and 40× magnification, the sinusoids in the areas of pallor (double-edged yellow arrows) contain large numbers of red blood cells (asterisk) and polymorphonuclear cells (arrowhead). The hepatocytes in the areas of pallor exhibit various features of cellular degeneration characterized by cytoplasmic vacuolation, nuclear pallor (black arrows), and nuclear pyknosis (yellow circles). (**B**)**.** Histologic sections of the duodenum from control and Elamipretide-treated pigs presented at various magnifications. At 2× magnification, the differences between control and treated pigs' duodenal sections are not readily apparent. At 10× magnification, the necrosis and sloughing of villous tips are evident in controls but not in the treated pigs (dashed yellow ellipse). At 40× magnification, individual enterocyte pallor is noticeable in the Elamipretide-treated pigs, but the villous tips are not sloughing. In contrast, in the control pig sections, the entire villous tips are sloughed. The enterocytes are necrotic (black arrows) and are admixed with polymorphonuclear cells (arrowheads). Also, capillary congestion is more prominent in the controls (black asterisk). Yellow stars indicate bacterial colonies.

The most striking findings were detected in the liver (Fig. 4A) and duodenum (Fig. 4B) section with light microscopy. Specifically, extensive areas of midzonal to centrilobular necrosis were observed in most of the control animals’ liver samples. In these areas, the hepatocytes were replaced by hemorrhage, or marked congestion was present in the sinusoids. Hepatocytes bordering the areas of sinusoidal hemorrhage exhibited cytocellular degenerative changes such as cytoplasmic vacuolation and blebbing. In duodenum sections, the enterocytes at the tips of the villi were either uniformly eosinophilic or sloughed (interpreted as necrosis). Additionally, increased numbers of polymorphonuclear cells were present in the affected areas in the interstitium of the lamina propria or the intestinal lumen. Although no statistical significance was detected, a clear trend of more severe changes was observed in the control samples. Microscopic changes were minimal in the sections of the myocardium and were primarily represented by shrinkage and eosinophilia of selected cardiomyocytes., Surprisingly no light microscopy-detectable changes were observed in the kidney sections. Importantly, kidneys from all animals treated with Elamipretide had no evident damage (injury score of 0). The histological grades are provided in Table 3.

**Table 3:** Light microscopy tissue injury grading. IQR: interquartile range.

Grade	Liver		Duodenum		Kidney		Heart	
	Elamipretide	Control	Elamipretide	Control	Elamipretide	Control	Elamipretide	Control
Median	1.5	0	0.5	1	0	0.5	0.5	1
IQR	1–3	0–2	0–2	1–3	0–0	0–1	0–1	0–3
*P* value	0.1	0.2	0.06	0.4				

**Figure 5:**
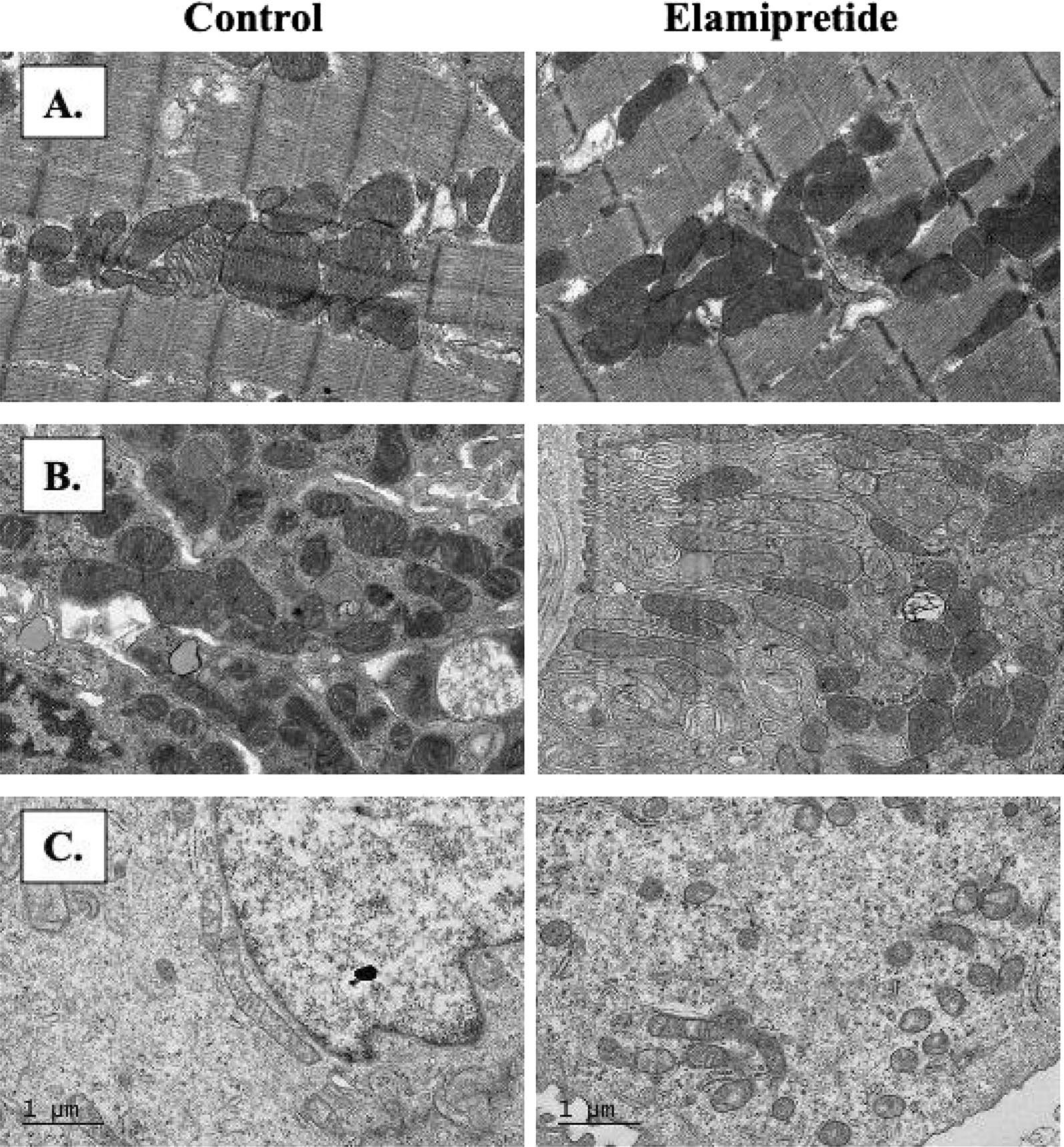
Electron microscopy images (4000x) of control and Elamipretide-treated pigs from the heart (**A**), renal cortex (**B**), and renal medulla (**C**). The mitochondrial shapes and architecture are preserved in the Elamipretide treated group.

On electron microscopy, there was no difference in mitochondrial injury (control: 1.5 [1.1–2.5]; Elamipretide: 1.3 ^1,2^; *p* = 0.4) or cell vacuolation (control: 1.25 ^1,2^; Elamipretide: 1 [0–1.5]; *p* = 0.5) between groups. However, there was a subjective improvement in mitochondrial morphology (shape and integrity) and preserved nuclear structure (Fig. 5).

## Discussion

The present study sought to mitigate IRI in a pig model of hemorrhagic shock and profound ischemia by using a peptide developed to protect mitochondrial function. We observed pleiotropic effects of Elamipretide. Namely, Elamipretide not only reduced the volume of isotonic crystalloids required to maintain normotension, but it also significantly reduced histologic and biochemical evidence of myocardial and renal injury.

. Elamipretide is a small peptide developed to augment mitochondrial function for patients with mitochondrial diseases^42^. The drug stabilizes the mitochondrial inner membrane by binding cardiolipin^35^, an abundant mitochondrial membrane phospholipid that exists in microdomains (*i.e.*, distinct membrane regions enriched in cardiolipin). The binding of Elamipretide to cardiolipin preserves mitochondrial cristae structure, improving energy production and reducing the production of damaging mitochondrial reactive oxygen species^35^. Several preclinical research models have shown that this peptide preserves mitochondrial function and provides cytoprotection. For instance, in vivo rabbit^38^, dog^36^, and sheep, as well as ex vivo guinea pig^43^ myocardial infarction studies, demonstrated a reduction in infarct size with Elamipretide treatment. Similarly, mice with ischemic strokes displayed reduced infarct size and improved neurological function following Elamipretide treatment^44^. In addition, studies in pigs have focused on establishing the benefits of Elamipretide in models of metabolic syndrome^45,46^ and atherosclerotic renal artery stenosis^47^. Most importantly, from a rapid translation standpoint, Elamipretide has already been tested in clinical trials for non-trauma indications, including heart failure^48,49^, an atherosclerotic renal stenosis Phase II trial^50^, an intermediate age-related macular degeneration Phase I trial^51^, and a Phase II–III trial for Barth syndrome, a genetic disorder of mitochondrial cardiolipin metabolism^52^. Yet, the mitochondrial protective effects of Elamipretide have not been thoroughly studied in the context of IRI, even though mitochondrial injury is an essential component of the pathophysiology, as seen in preclinical trauma models. For example, a study utilizing a porcine polytrauma model (hemorrhagic shock with liver laceration, pulmonary contusions, and tibial fracture) demonstrated hepatic mitochondrial injury by electron microscopy and was associated with mitochondrial autophagy, cell apoptosis, and recruitment of anti-inflammatory leukocytes to the liver^53^.

Patients *in extremis* who received lifesaving maneuvers like REBOA will suffer a more severe mitochondrial dysfunction when compared to more traditional resuscitation practices for hemorrhagic shock. The exacerbated IRI is driven by complete or partial aortic occlusion, which likely worsens mitochondrial injury. This injury could explain the profound vasodilation, and distributive shock state often observed in those patients, even after their circulating blood volume is restored with transfusion and volume resuscitation. In addition, this severe IRI drives the need for large ongoing resuscitation requirements and significant vasopressor support, both increasing complications such as acute kidney and lung injury^54^. Therefore, pharmacological interventions that reduce mitochondrial dysfunction in trauma patients with severe IRI may improve morbidity and mortality by influencing the patient's response to resuscitation.

In the present study, animals treated with Elamipretide required less crystalloid for resuscitation than controls. This is significant because early hemorrhagic shock resuscitation with primarily saline-based regimens is linked to greater mortality, reduced cardiac output, and cardiac dysfunction^54^. Large-volume crystalloid resuscitation can lead to many complications, or resuscitation injuries, to various organs, such as the kidney, liver, and intestinal tract^11–13^. It promotes cell edema, dysfunction, and, ultimately, death^13,54^. In a study using a rat model of hemorrhagic shock, overly aggressive fluid resuscitation induced hepatocellular injury^55^. Furthermore, they found excessively aggressive fluid resuscitation was no better than more conservative resuscitation approaches at correcting plasma lactate concentration and preserving renal function. Overall, the study showed that large-volume isotonic crystalloid resuscitation provided no overall survival benefit. Crystalloid resuscitation and the associated dilution of coagulation factors also perpetuate the dysregulated coagulation state often observed after severe trauma^15–17^. In fact, hemodilution is one of the six main precipitants of coagulopathy in trauma (tissue trauma, shock, hemodilution, hypothermia, acidemia, and inflammation)^56^. Large-volume crystalloid resuscitation promotes interstitial edema^14^, a significant risk for abdominal compartment syndrome, a complication with a mortality rate > 50%^57^. Reducing fluid requirements (Fig. 4B) will also benefit resuscitation in resource-limited environments such as remote rural areas or military environments. Based on our data, in an average 70 kg pig, Elamipretide reduced fluid requirements by 2.6 L [1.8–4.4] liters. This reduction in fluid requirement may be due to preserved vascular motor tone. While the effects of Elamipretide on large arteries and peripheral arterioles have not been described, Elamipretide promotes blood flow to the brain via nitric oxide-mediated cerebromicrovascular dilation^58^. Since there was no difference in norepinephrine in conjunction with a reduction in fluid requirements, Elamipretide may preserve capillary integrity and vascular responsiveness to endogenous and exogenous vasopressors. This particular effect of Elamipretide may be attributed to mitochondrial function protection within vascular smooth muscle cells. Yet, several other mechanisms might contribute. In addition to direct action on smooth muscle metabolism in response to injury, it is plausible that Elamipretide might stimulate the sympathetic system, thereby promoting vasoconstriction^59^. This finding could be a direct effect that has not been previously described and may be mediated by other pathways, such as the IL-6 pathway. Conversely, Elamipretide might downregulate vasodilatory pathways (likely through the downregulation of nitric oxide production). Finally, maintaining endothelial integrity via metabolic protection reduces fluid losses in the interstitium through capillary leakage. It is possible that the reduction in interleukin 6 at the end of the experiment in the Elamipretide group is the result of inflammation downregulation, which could be mediated by activation of cholinergic anti-inflammatory reflex pathways^60–62^ or a direct effect of Elamipretide on interleukin 6 expression. Other studies of REBOA in pigs have demonstrated an elevation in serum interleukin 6 concentration^63–65^, which correlated with injury severity in trauma patients^66^. Interleukin 6 expression is associated with tissue injury, especially to the liver, and is directly related to mitochondrial injury^67–69^. In reality, multiple mechanisms of action likely influenced different organ systems in ways that reduced the resuscitation requirements we observed.

In the specific context of REBOA as an adjunct for shock resuscitation, Elamipretide had protective effects not only on tissue beds below the level of occlusion but also on organs proximal to the level of occlusion. Myocardial injury is a common sequela of REBOA and is likely related to increased work strain against increased afterload due to occlusion. This strain on the heart can be mitigated via partial occlusion^70^ or pharmacological intervention with beta-blockers to protect cardiomyocytes and reduce myocardial work^71^. In the present study, we have indications of a different pharmacologic method to reduce myocardial injury with Elamipretide. This protective effect likely translated to improved myocardial function, leading to the observed hemodynamic homeostasis in the treatment group during the critical care phase with no difference in serum lactate concentration, despite a much smaller volume of crystalloids administered. Similarly we observed that Elamipretide promoted renal protection homeostasis as evidenced by a reduction in serum creatinine concentration. We believe this finding results from direct mitochondrial protection in the kidney, improved oxygen delivery to the kidney, or a combination of both.

This study is subject to the limitations that characterize most large animal translational studies. First, we followed the animals for a limited duration, which may not be sufficient to replicate delayed complications observed in severely traumatized patients. This may also explain why the changes observed on histopathology and electron microscopy were not as pronounced as they might have been after a more extended study. Rigorous preclinical studies are crucial to ensure successful translation to humans. Guidelines have outlined the preclinical path to minimize the risk of translational failure^72^. Second, we performed a controlled hemorrhage without additional significant tissue damage; a polytrauma model (pulmonary contusion, long bone fracture, traumatic brain injury, burn, etc.) may reflect more realistic injuries such as those observed in patients who are so critically injured; they require additional lifesaving maneuvers like REBOA. Third, inhaled anesthetics may protect against IRI, and studies with intravenous anesthesia should be conducted to verify our findings^73^ Finally, despite our randomization efforts, there were statistically significant differences between groups in MAP at baseline, likely a type I error. While the differences were significant statistically, their biological relevance is probably limited. Interindividual variability in response to surgical setup may have contributed to some of our findings. Our group has already highlighted the unexpected variability in response to a similar model^74^. Regardless, blood pressure alone is not a good marker of microperfusion overall. Differences of that magnitude are unlikely to account for our findings in a model of such profound IRI. Larger studies are needed to address the above points.

Those limitations notwithstanding, the present data demonstrate that Elamipretide reduced fluid requirements and protected the heart and kidney in a pig model of hemorrhage and REBOA. Those benefits support our hypothesis that pharmacological interventions targeting mitochondria will likely benefit trauma patients with severe IRI. Future studies will focus on elucidating the mechanisms behind our observed benefits with a focus on cardiovascular and renal cellular metabolism as well as evaluating mitochondrial function in various tissue beds.

## Supplementary Information

Supplementary Figure 2.

Supplementary Figure 2.

Supplementary Figure 3.

Supplementary Legends.

## Data Availability

The datasets during and/or analyzed during the current study are available from the corresponding author upon reasonable request.

## References

[1] Slottosch I (2014). Controlled lung reperfusion to reduce pulmonary ischaemia/reperfusion injury after cardiopulmonary bypass in a porcine model. Interact. Cardiovasc. Thorac. Surg..

[2] Durrani NK (2006). The effect of gradually increased blood flow on ischemia-reperfusion injury in rat kidney. Am. J. Surg..

[3] Xu WW (2018). Ischemia reperfusion injury after gradual versus rapid flow restoration for middle cerebral artery occlusion rats. Sci. Rep..

[4] Obert DP, Wolpert AK, Korff S (2019). Modulation of endoplasmic reticulum stress influences ischemia-reperfusion injury after hemorrhagic shock. Shock.

[5] Heusch G (2020). Myocardial ischaemia-reperfusion injury and cardioprotection in perspective. Nat. Rev. Cardiol..

[6] Heusch G (2015). Molecular basis of cardioprotection: signal transduction in ischemic pre-, post-, and remote conditioning. Circ. Res..

[7] Kleinbongard P, Skyschally A, Heusch G (2017). Cardioprotection by remote ischemic conditioning and its signal transduction. Pflugers Arch..

[8] Bell RM (2022). Remote ischaemic conditioning: defining critical criteria for success-report from the 11th Hatter Cardiovascular Workshop. Basic Res. Cardiol..

[9] Kloner RA, Shi J, Dai W, Carreno J, Zhao L (2020). Remote ischemic conditioning in acute myocardial infarction and shock states. J. Cardiovasc. Pharmacol. Ther..

[10] Huang J (2018). Remote ischemic postconditioning improves myocardial dysfunction via the risk and safe pathways in a rat model of severe hemorrhagic shock. Shock.

[11] Lu YQ (2007). Experimental study of controlled fluid resuscitation in the treatment of severe and uncontrolled hemorrhagic shock. J. Trauma.

[12] Wang L, Pei F, Wu J, Ouyang B, Guan X (2021). Kidney injury in a hemodilution model of hemorrhagic shock and fluid resuscitation. Am. J. Med. Sci..

[13] Chatrath V, Khetarpal R, Ahuja J (2015). Fluid management in patients with trauma: restrictive versus liberal approach. J. Anaesthesiol. Clin. Pharmacol..

[14] Balogh Z (2003). Supranormal trauma resuscitation causes more cases of abdominal compartment syndrome. Arch. Surg..

[15] Nishi K, Takasu A, Shinozaki H, Yamamoto Y, Sakamoto T (2013). Hemodilution as a result of aggressive fluid resuscitation aggravates coagulopathy in a rat model of uncontrolled hemorrhagic shock. J. Trauma Acute Care Surg..

[16] Bolliger D, Szlam F, Levy JH, Molinaro RJ, Tanaka KA (2010). Haemodilution-induced profibrinolytic state is mitigated by fresh-frozen plasma: implications for early haemostatic intervention in massive haemorrhage. Br. J. Anaesth..

[17] Bolliger D (2009). Finding the optimal concentration range for fibrinogen replacement after severe haemodilution: an in vitro model. Br. J. Anaesth..

[18] Causey MW, Salgar S, Singh N, Martin M, Stallings JD (2012). Valproic acid reversed pathologic endothelial cell gene expression profile associated with ischemia-reperfusion injury in a swine hemorrhagic shock model. J. Vasc. Surg..

[19] Nie C (2021). Hydrogen gas inhalation alleviates myocardial ischemia-reperfusion injury by the inhibition of oxidative stress and NLRP3-mediated pyroptosis in rats. Life Sci..

[20] Cannistrà M (2016). Hepatic ischemia reperfusion injury: a systematic review of literature and the role of current drugs and biomarkers. Int. J. Surg..

[21] Biesterveld BE (2021). Valproic acid protects against acute kidney injury in hemorrhage and trauma. J. Surg. Res..

[22] Liu FC, Tsai YF, Tsai HI, Yu HP (2015). Anti-inflammatory and organ-protective effects of resveratrol in trauma-hemorrhagic injury. Med. Inflamm..

[23] Hilbert-Carius P, McGreevy DT, Abu-Zidan FM, Hörer TM (2020). Pre-hospital CPR and early REBOA in trauma patients - results from the ABOTrauma registry. World J. Emerg. Surg..

[24] Bini JK (2022). Survival benefit for pelvic trauma patients undergoing resuscitative endovascular balloon occlusion of the aorta: results of the aast aortic occlusion for resuscitation in trauma acute care surgery (AORTA) registry. Injury.

[25] Kuckelman J (2019). Efficacy of intermittent versus standard resuscitative endovascular balloon occlusion of the aorta in a lethal solid organ injury model. J. Trauma Acute Care Surg..

[26] Johnson MA (2020). Not ready for prime time: Intermittent versus partial resuscitative endovascular balloon occlusion of the aorta for prolonged hemorrhage control in a highly lethal porcine injury model. J. Trauma Acute Care Surg..

[27] Gorman E (2021). High resuscitative endovascular balloon occlusion of the aorta procedural volume is associated with improved outcomes: an analysis of the AORTA registry. J. Trauma Acute Care Surg..

[28] Causey MW (2013). Beneficial effects of histone deacetylase inhibition with severe hemorrhage and ischemia-reperfusion injury. J. Surg. Res..

[29] Zhang M (2013). Dynamic change of hydrogen sulfide after traumatic brain injury and its effect in mice. Neurochem. Res..

[30] Conner J (2021). Combatting ischemia reperfusion injury from resuscitative endovascular balloon occlusion of the aorta using adenosine, lidocaine and magnesium: a pilot study. J. Trauma Acute Care Surg..

[31] Cairns CB (1997). Evidence for early supply independent mitochondrial dysfunction in patients developing multiple organ failure after trauma. J. Trauma.

[32] Aswani A (2018). Scavenging circulating mitochondrial DNA as a potential therapeutic option for multiple organ dysfunction in trauma hemorrhage. Front. Immunol..

[33] Villarroel JP (2013). Hemorrhagic shock and resuscitation are associated with peripheral blood mononuclear cell mitochondrial dysfunction and immunosuppression. J. Trauma Acute Care Surg..

[34] Hellberg A (2001). Monitoring of intrathecal oxygen tension during experimental aortic occlusion predicts ultrastructural changes in the spinal cord. J. Thorac. Cardiovasc. Surg..

[35] Allen ME (2020). The cardiolipin-binding peptide elamipretide mitigates fragmentation of cristae networks following cardiac ischemia reperfusion in rats. Commun. Biol..

[36] Sabbah HN (2016). Chronic therapy with elamipretide (MTP-131), a novel mitochondria-targeting peptide, improves left ventricular and mitochondrial function in dogs with advanced heart failure. Circ. Heart Fail..

[37] Eirin A (2014). Mitochondrial protection restores renal function in swine atherosclerotic renovascular disease. Cardiovasc. Res..

[38] Brown DA (2014). Reduction of early reperfusion injury with the mitochondria-targeting peptide bendavia. J. Cardiovasc. Pharmacol. Ther..

[39] Eirin A (2019). Urinary mitochondrial DNA copy number identifies renal mitochondrial injury in renovascular hypertensive patients undergoing renal revascularization: a Pilot Study. Acta Physiol. (Oxf).

[40] Shults NV, Kanovka SS, Ten Eyck JE, Rybka V, Suzuki YJ (2019). Ultrastructural changes of the right ventricular myocytes in pulmonary arterial hypertension. J. Am. Heart Assoc..

[41] Kellum JA, Lameire N (2013). Diagnosis, evaluation, and management of acute kidney injury: a KDIGO summary (Part 1). Crit. Care.

[42] Obi C, Smith AT, Hughes GJ, Adeboye AA (2022). Targeting mitochondrial dysfunction with elamipretide. Heart Fail. Rev..

[43] Kloner RA (2012). Reduction of ischemia/reperfusion injury with bendavia, a mitochondria-targeting cytoprotective Peptide. J. Am. Heart Assoc..

[44] Imai T, Matsubara H, Nakamura S, Hara H, Shimazawa M (2020). The mitochondria-targeted peptide, bendavia, attenuated ischemia/reperfusion-induced stroke damage. Neuroscience.

[45] Yuan F (2018). Mitoprotection attenuates myocardial vascular impairment in porcine metabolic syndrome. Am. J. Physiol. Heart Circ. Physiol..

[46] Eirin A (2018). Mitoprotection preserves the renal vasculature in porcine metabolic syndrome. Exp. Physiol..

[47] Kim SR, Eirin A, Zhang X, Lerman A, Lerman LO (2019). Mitochondrial protection partly mitigates kidney cellular senescence in swine atherosclerotic renal artery stenosis. Cell Physiol. Biochem..

[48] Chatfield KC (2019). Elamipretide improves mitochondrial function in the failing human heart. JACC Basic Transl. Sci..

[49] Butler J (2020). Effects of elamipretide on left ventricular function in patients with heart failure with reduced ejection fraction: the PROGRESS-HF phase 2 trial. J. Card. Fail..

[50] Szeto HH (2017). Pharmacologic approaches to improve mitochondrial function in AKI and CKD. J. Am. Soc. Nephrol..

[51] Allingham MJ, Mettu PS, Cousins SW (2022). Phase 1 clinical trial of elamipretide in intermediate age-related macular degeneration and high-risk drusen: ReCLAIM high-risk drusen study. Ophthalmol. Sci..

[52] Reid Thompson W (2021). A phase 2/3 randomized clinical trial followed by an open-label extension to evaluate the effectiveness of elamipretide in Barth syndrome, a genetic disorder of mitochondrial cardiolipin metabolism. Genet. Med..

[53] Shi Y (2021). Trauma-hemorrhage stimulates immune defense, mitochondrial dysfunction, autophagy, and apoptosis in pig liver at 72 h. Shock.

[54] Cotton BA, Guy JS, Morris JA, Abumrad NN (2006). The cellular, metabolic, and systemic consequences of aggressive fluid resuscitation strategies. Shock.

[55] Shah KJ, Chiu WC, Scalea TM, Carlson DE (2002). Detrimental effects of rapid fluid resuscitation on hepatocellular function and survival after hemorrhagic shock. Shock.

[56] Hess JR (2008). The coagulopathy of trauma: a review of mechanisms. J. Trauma.

[57] Kirkpatrick AW (2013). Intra-abdominal hypertension and the abdominal compartment syndrome: updated consensus definitions and clinical practice guidelines from the World Society of the Abdominal Compartment Syndrome. Intensive Care Med..

[58] Tarantini S (2018). Treatment with the mitochondrial-targeted antioxidant peptide SS-31 rescues neurovascular coupling responses and cerebrovascular endothelial function and improves cognition in aged mice. Aging Cell.

[59] März P (1998). Sympathetic neurons can produce and respond to interleukin 6. Proc. Natl. Acad. Sci. U S A.

[60] Kelly MJ, Breathnach C, Tracey KJ, Donnelly SC (2022). Manipulation of the inflammatory reflex as a therapeutic strategy. Cell Rep. Med..

[61] Deng J, Jiang Y, Wang M, Shao L, Deng C (2021). Activation of vagovagal reflex prevents hepatic ischaemia-reperfusion-induced lung injury via anti-inflammatory and antioxidant effects. Exp. Physiol..

[62] Lieder HR (2018). Vago-splenic axis in signal transduction of remote ischemic preconditioning in pigs and rats. Circ. Res..

[63] Sadeghi M (2018). Blood pressure targeting by partial REBOA is possible in severe hemorrhagic shock in pigs and produces less circulatory, metabolic and inflammatory sequelae than total REBOA. Injury.

[64] Morrison JJ (2014). Use of resuscitative endovascular balloon occlusion of the aorta in a highly lethal model of noncompressible torso hemorrhage. Shock.

[65] Sadeghi M (2020). Total resuscitative endovascular balloon occlusion of the aorta causes inflammatory activation and organ damage within 30 minutes of occlusion in normovolemic pigs. BMC Surg..

[66] Okeny PK, Ongom P, Kituuka O (2015). Serum interleukin-6 level as an early marker of injury severity in trauma patients in an urban low-income setting: a cross-sectional study. BMC Emerg. Med..

[67] Yang R (2003). IL-6 is essential for development of gut barrier dysfunction after hemorrhagic shock and resuscitation in mice. Am. J. Physiol. Gastrointest Liver Physiol..

[68] Toth B (2004). Insights into the role of interleukin-6 in the induction of hepatic injury after trauma-hemorrhagic shock. J. Appl. Physiol..

[69] Kielar ML (2005). Maladaptive role of IL-6 in ischemic acute renal failure. J. Am. Soc. Nephrol..

[70] Beyer CA (2019). Resuscitative endovascular balloon occlusion of the aorta induced myocardial injury is mitigated by endovascular variable aortic control. J. Trauma Acute Care Surg..

[71] Hoareau GL (2020). Esmolol reduces myocardial injury induced by resuscitative endovascular balloon occlusion of the aorta (REBOA) in a porcine model of hemorrhagic shock. Injury.

[72] Lecour S (2021). IMproving Preclinical Assessment of Cardioprotective Therapies (IMPACT) criteria: guidelines of the EU-CARDIOPROTECTION COST Action. Basic Res. Cardiol..

[73] Kleinbongard P, Bøtker HE, Ovize M, Hausenloy DJ, Heusch G (2020). Co-morbidities and co-medications as confounders of cardioprotection-Does it matter in the clinical setting?. Br. J. Pharmacol..

[74] Martin SC (2022). Unmasking the confounder: the inherent physiologic variability of swine during an automated experimental model of ischemia-reperfusion injury. Am. Surg..

