# Supplementary figures and images for: Elamipretide mitigates ischemia-reperfusion injury in a swine model of hemorrhagic shock
Source: Sci Rep. 2023 Mar 18;13:4496. doi: 10.1038/s41598-023-31374-5 (PMC10024723; doi:10.1038/s41598-023-31374-5)

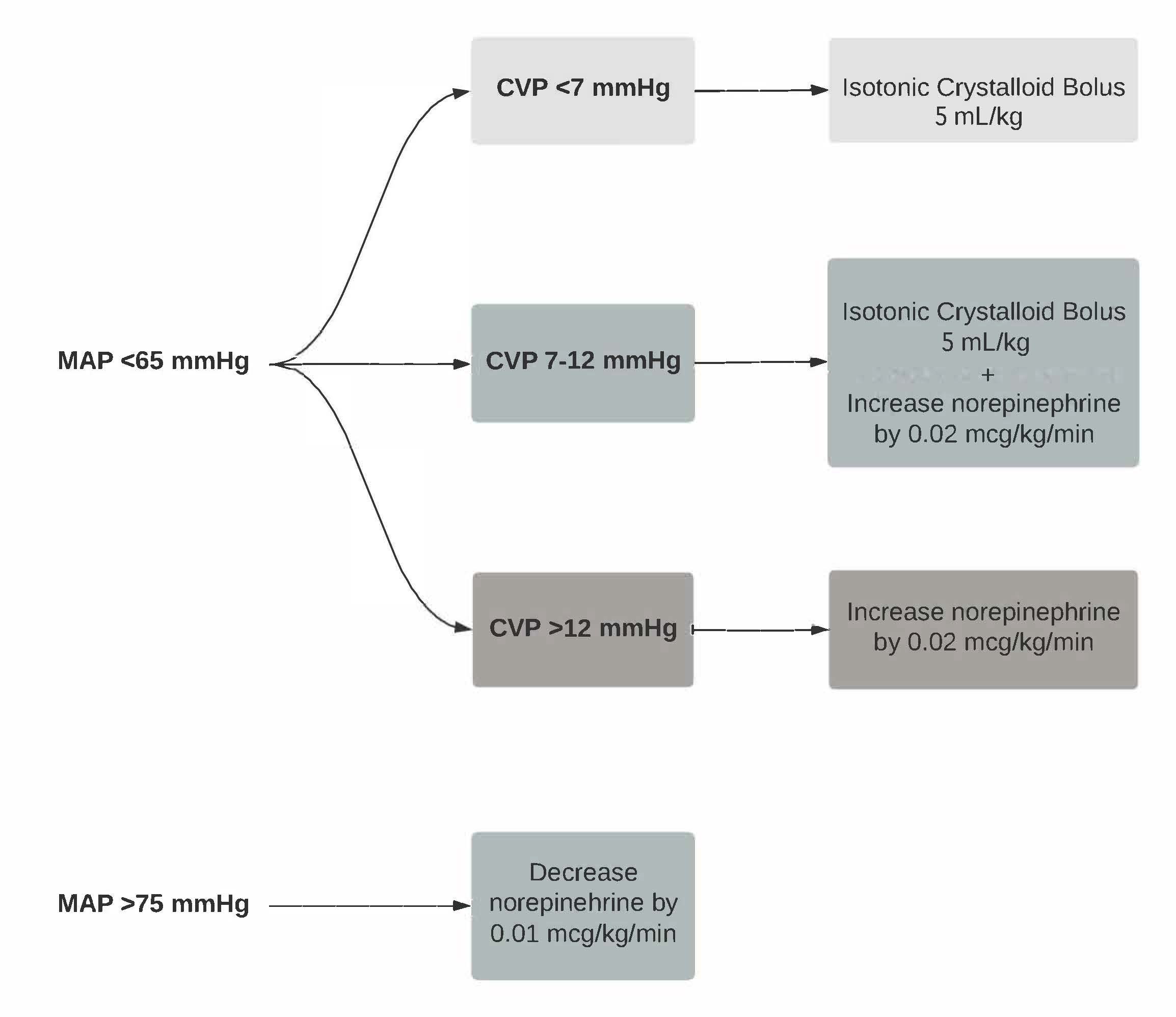

Supplement: Supplementary file 1 — Supplementary Figure 2. [file 41598_2023_31374_MOESM1_ESM.jpg]

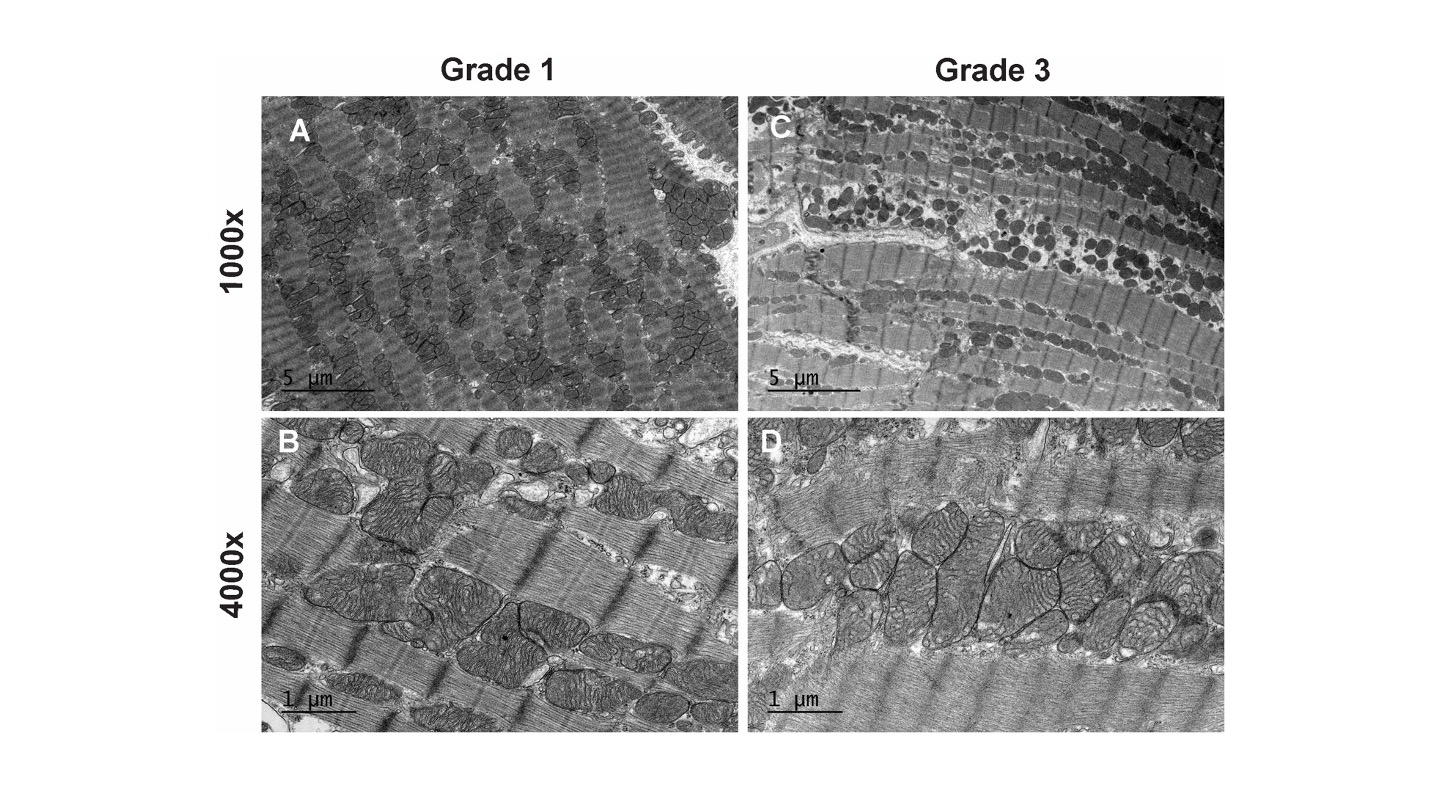

Supplement: Supplementary file 2 — Supplementary Figure 2. [file 41598_2023_31374_MOESM2_ESM.jpg]

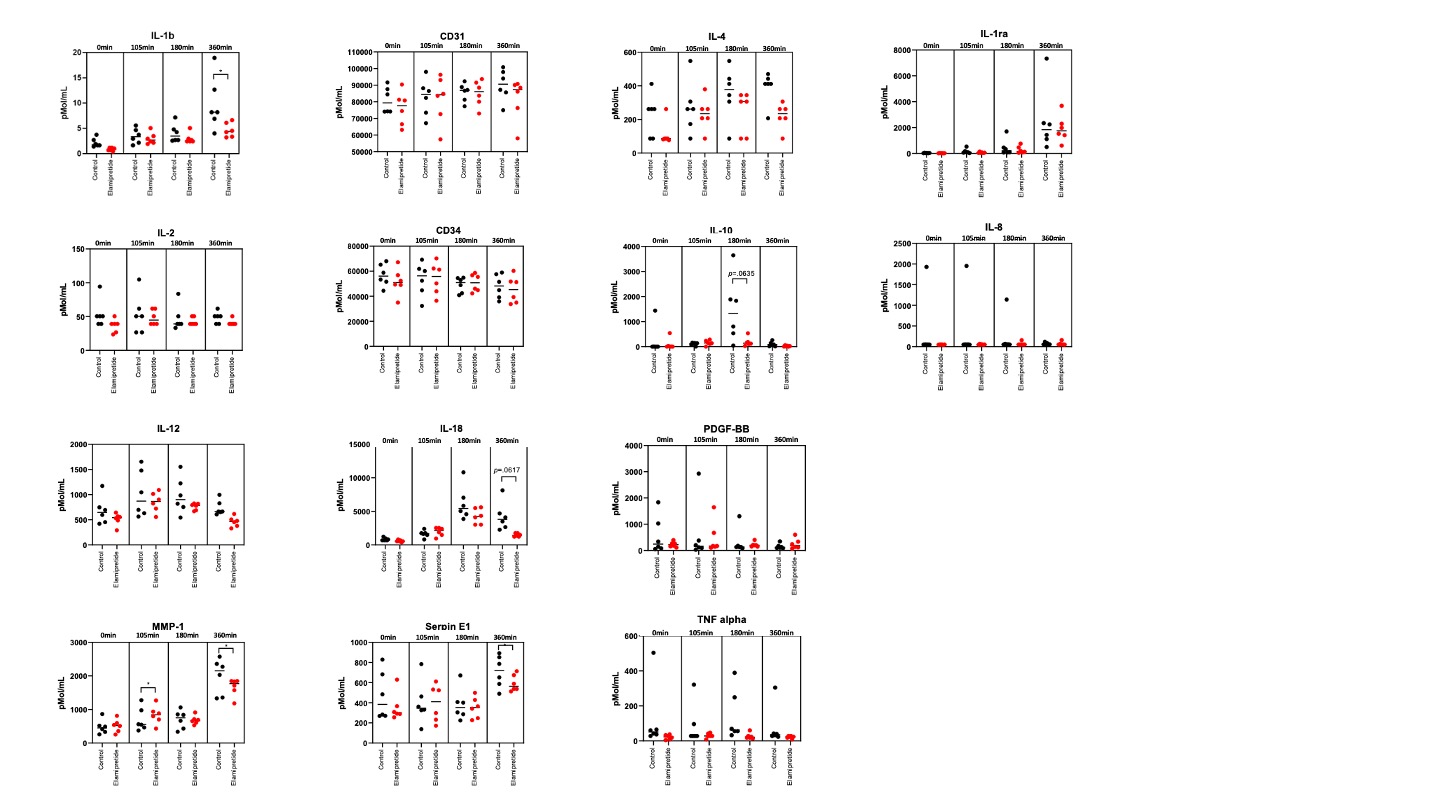

Supplement: Supplementary file 3 — Supplementary Figure 3. [file 41598_2023_31374_MOESM3_ESM.jpg]
