# Supplementary material for: Elamipretide mitigates ischemia-reperfusion injury in a swine model of hemorrhagic shock
Source: Sci Rep. 2023 Mar 18;13:4496. doi: 10.1038/s41598-023-31374-5 (PMC10024723; doi:10.1038/s41598-023-31374-5)
Supplement: Supplementary file 4 — Supplementary Legends. [file 41598_2023_31374_MOESM4_ESM.docx]

**Supplemental Figure 1.** Critical Care Algorithm.

**Supplemental Figure 2.** Example of heart transmission electron microscopy images at 1000x and 4000x magnifications. **A.** Note that at grade 1, mitochondria are densely packed and have prominent intermitochondrial junctions (IMJ). **B.** Same specimens at 4000x. The IMJs are still present, but there are some minimal clear spaces within and around the mitochondria. Cristae are properly aligned and intact. **C.** In Grade 3 there is abundant clear space surrounding the mitochondria, indicative of cellular edema. **D.** The mitochondrial cristae are swollen, rounded, or indistinct. The IMJs are smudged. The Z-bands are electron-loos,e and the myofibers are disrupted in some areas.

**Supplemental Figure 3.** Individual cytokine serum concentrations over time.
